# Applying existing clinical staging models in a sample of Italian bipolar patients over a 10-years follow-up

**DOI:** 10.1192/j.eurpsy.2022.424

**Published:** 2022-09-01

**Authors:** M. Macellaro, N. Girone, L. Cremaschi, M. Bosi, B. Cesana, F. Ambrogi, B. Dell’Osso

**Affiliations:** 1 University of Milan, Department Of Mental Health, Department Of Biomedical And Clinical Sciences Luigi Sacco, Milan, Italy; 2 University of Milan, Department Of Clinical Sciences And Community Health, Unit Of Medical Statistics, Biometrics And Bioinformatics “giulio A. Maccacaro”, Faculty Of Medicine And Surgery, Milan, Italy; 3 University of Milan, “aldo Ravelli” Center For Nanotechnology And Neurostimulation, Milan, Italy; 4 Department of Psychiatry and Behavioral Sciences, Stanford University, Milan, Italy

**Keywords:** bipolar disorder, Staging Models

## Abstract

**Introduction:**

Bipolar Disorder (BD) is a life-course illness with evidence of a progressive nature. Although different staging models have been proposed from a theoretical perspective,longitudinal studies are scarce.

**Objectives:**

The aim of the present study was to apply four staging models in a sample of BD patients and to observe their progression in 10 years of retrospective evaluation.

**Methods:**

In a naturalistic sample of 100 BD patients, a retrospective assessment of clinical stages across 10 years of observation at six time points (T0: 2010; T1: 2013; T2: 2015; T3: 2018; T4: 2019; T5:2020) was performed according to the BD staging models (Berk et al., 2007; Kapczinski et al., 2009; Kupka et al., 2012 and Duffy et al., 2014). Socio-demographic and clinical variables were collected and the staging progression across time was analyzed.

**Results:**

A significant progressive staging worsening emerged over 10 years of BD observation for each examined model (p<0.001). Moreover, for all considered staging approaches, stage values were lower over the time points for BD II, lower number of lifetime episodes and hospitalizations (p<0.05). Finally, the stage increase was associated with a lower age at first elevated episode (p<0.05).

**Conclusions:**

Present preliminary results confirm the relevance of illness onset and early intervention in BD, given their role in patients classified into worse clinical staging. There is an emerging need of a standardized universal staging model in order to better characterize BD patients, their treatment and their clinical course.

**Disclosure:**

No significant relationships.

